# Smart clothes-assisted home-nursing care program for family caregivers of older persons with dementia and hip fracture: a mixed-methods study

**DOI:** 10.1186/s12877-022-02789-y

**Published:** 2022-02-05

**Authors:** Yi-Jun Hou, Sih-Ying Zeng, Chung-Chih Lin, Ching-Tzu Yang, Huei-Ling Huang, Min-Chi Chen, Hsiu-Hsin Tsai, Jersey Liang, Yea-Ing L. Shyu

**Affiliations:** 1 Department of Nursing, Chang Gung Memorial Hospital, Taoyuan, Taiwan; 2 Department of Nursing, Far Eastern Memorial Hospital, Banciao, Taiwan; 3 Department of Computer Science and Information Engineering, Chang Gung University, Taoyuan, Taiwan; 4 School of Nursing, Chang Gung University, 259 Wenhua 1st Road, Guishan District, Taoyuan, 33302 Taiwan; 5 Department of Gerontology and Health Care Management, Chang Gung University of Science and Technology, Taoyuan, Taiwan; 6 Department of Public Health and Parasitology, Chang Gung University, Taoyuan, Taiwan; 7 School of Public Health, University of Michigan, Ann Arbor, MI USA; 8 Institute of Gerontology, University of Michigan, Ann Arbor, MI USA; 9 Dementia Center, Department of Neurology, Chang Gung Memorial Hospital, Taoyuan, Taiwan; 10 Healthy Aging Research Center, Chang Gung University, Taoyuan, Taiwan; 11 Department of Nursing, Kaohsiung Chang Gung Memorial Hospital, Kaohsiung, Taiwan

**Keywords:** Dementia, Family caregivers, Hip fracture, Homecare nursing, Smart clothes

## Abstract

**Background:**

The purpose of this preliminary study was to explore whether a smart clothes-assisted home-nursing care program could benefit family caregivers and their care recipients.

**Methods:**

Family caregivers in charge of a care recipient’s living situation participated in this convergent parallel, mixed methods study. We recruited older persons with dementia (*n* = 7) and those discharged following hip-fracture surgery (*n* = 6) from neurological clinics and surgical wards of a medical center, respectively, along with their family caregivers: three spouses, eight sons, one daughter, and one daughter-in-law. Care recipients were asked to wear a smart vest at least 4 days/week for 6 months, which contained a coin-size monitor hidden in an inner pocket. Sensors installed in bedrooms and living areas received signals from the smart clothing, which were transmitted to a mobile phone app of homecare nurses, who provided caregivers with transmitted information regarding activities, emergency situations and suggestions for caregiving activities. Outcomes included changes from baseline in caregivers’ preparedness and depressive symptoms collected at 1- and 3-months, which were analyzed with Friedman’s non-parametric test of repeated measures with post-hoc analysis. Transcripts of face-to-face semi-structured interview data about caregivers’ experiences were analyzed to identify descriptive, interpretative, and pattern codes.

**Results:**

Preparedness did not change from baseline at either 1- or 3-months for family caregivers of persons with dementia. However, depressive symptoms decreased significantly at 1-month and 3-months compared with baseline, but not between 1-months and 3-months. Analysis of the interview data revealed the smart clothes program increased family caregivers’ knowledge of the care recipient’s situation and condition, informed healthcare providers of the care recipient’s physical health and cognitive status, helped homecare nurses provide timely interventions, balanced the care recipient’s exercise and safety, motivated recipients to exercise, helped family caregivers balance work and caregiving, and provided guidance for caregiving activities.

**Conclusions:**

Experiences with the smart clothes-assisted home-nursing care program directly benefited family caregivers, which provided indirect benefits to the care recipients due to the timely interventions and caregiving guidance from homecare nurses. These benefits suggest a smart-clothes-assisted program might be beneficial for all family caregivers.

## Background

Family caregivers of older persons with a physical or cognitive disability experience caregiver burden, depressive symptoms, sleep disturbances, which impact health outcomes and health-related quality of life [25]. The impact of depressive symptoms is greatest for these caregivers as it significantly increases the risk of depression compared with caregivers with no depressive symptoms [10, 18]. Caregiver depression has been associated with the older care recipient’s levels of cognitive impairment, self-care ability, and neuropsychiatric symptoms [6, 12, 13, 26]. Approximately one-third of family caregivers of persons living with dementia have depression [8]. Smart care systems, which integrates technology with online or mobile services, has been developed to support these family caregivers [1]. However, few studies have examined the benefits of a smart care-based home nursing program.

Wireless technologies with noninvasive sensors have been integrated into clothing to enhance collecting biomedical data to monitor a wearer’s physiological parameters and prevent disease and enhance adherence to rehabilitation [2, 21, 24]. Smart care, which involves environmental and wearable sensors that transmit information from the home to a health care setting, and remote health technology have been developed to facilitate family caregiving at home for older persons with physical or cognitive impairment [1, 3]. However, smart technologies have made only modest contributions to supporting caregivers, with little empirical evidence to support their effectiveness [1].

Different from the prior smart homecare model, the smart care in this study combined smart clothes technology with home nursing care and targeted caregivers of older persons with a cognitive or physical disability. The smart clothes-assisted home-nursing care (SCA home-nursing care) program was implemented to facilitate family caregiving to older persons with dementia and those recovering from hip-fracture surgery. The homecare nurses provide family caregivers guidance and support based on information transmitted by home sensors from the smart clothes. Therefore, the purpose of this preliminary study was to explore the experiences of family caregivers receiving the SCA home-nursing care program and whether it benefited their caregiving and their care recipients.

## Methods

### Study design

A convergent parallel, mixed-methods design, i.e., concurrent qualitative and quantitative approaches in the same phase of the research process, was implemented at a medical center in northern Taiwan from July 2018 to September 2019. In this mixed-methods approach, the quantitative and qualitative components were equally implemented, analyzed independently, and interpreted together [9].

### Participants

A convenience sample of family caregivers of older persons with dementia or recovering from hip-fracture surgery was recruited from a medical center in northern Taiwan. Research nurses identified caregivers of patients who met the research criteria as potential participants and provided an explanation of the design and purpose of the study. Interested caregivers were invited to participate; those who agreed provided signed informed consent.

Patients were included by these criteria: 1) age 60 years or older; 2) diagnosed with dementia or received surgery for a hip fracture; 3) could walk independently or with assistance; 4) receiving care from a family member related by kinship; and 5) living in northern Taiwan. Exclusion criteria were 1) with psychiatric disease; 2) terminally ill; (3) without a primary family caregiver, and (4) living in an institution. Family caregivers were included by these criteria: (1) ≥ 20 years of age, and (2) responsible for providing direct care or supervising the care received by the patient.

### Smart clothes-assisted (SCA) home-nursing care

The smart clothes used in this study were designed and developed by C. C. Lin. They have been implemented in nursing homes and are available commercially [16]. However, the application of smart clothes in a home setting with family caregivers who provide care to persons with dementia, or a physical disability has not been examined.

**Fig. 1:**
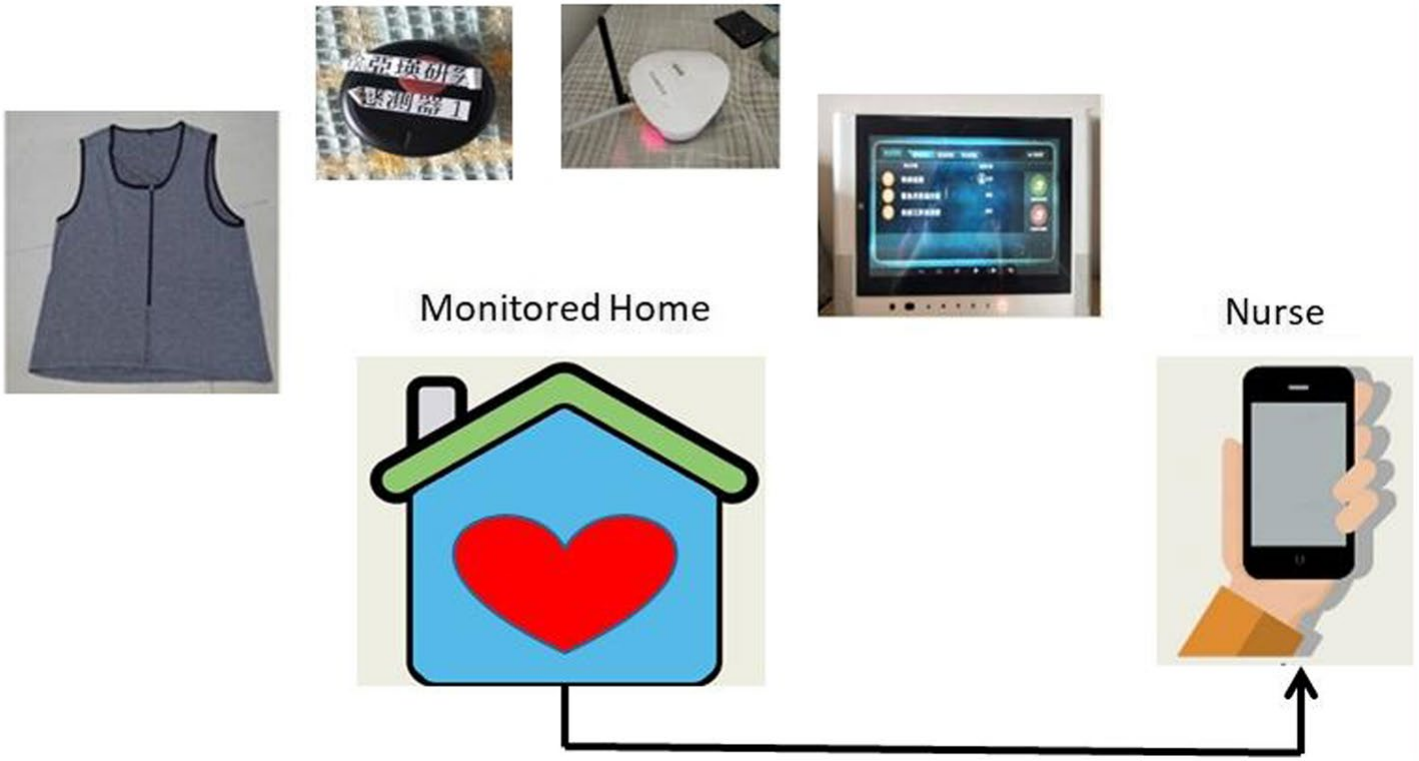
Smart clothes-assisted (SCA) home-nursing care program

Therefore, the model of the SCA home-nursing care program was based on the use of the smart clothing combined with a remote monitoring system (Fig. 1), to assist family caregivers of persons with dementia or recovering from hip-fracture surgery. Care recipients were asked to wear a smart vest, which contained a coin-sized monitor hidden in an inner pocket. The smart clothes monitor recorded activity levels of the care recipients, which was measured by the number of steps taken per day as well as any periods of inactivity. Sensors were installed in bedrooms and living areas to receive signals from the smart clothing about location; an emergency button and smoke detector was installed in all homes. An alarm was installed at doors leading outside for persons with dementia to prevent wandering. The sensors transmitted signals and emergency information to homecare nurses responsible for overseeing program, via an app installed on their mobile phones. Signals received on the mobile phone by the homecare nurse were used to provide feedback to family caregivers about the care recipients activities, which included emergency calls, frequency of getting up at night, staying in the bathroom for more than 30 min, inadequate or abnormal activity levels, not moving during the day for more than 2 h, leaving the house alone, and a sensor being disconnected from the system. Based on information and signals from the smart-care sensors, homecare nurses discussed caregiving activities and planning with family caregivers.

Care recipients were asked to the wear smart clothing for at least 4 days/week for 6 months. A homecare research nurse visited the home setting to assess it for sensor installation and to suggest environmental modifications for potential hazards. A second visit was made by the research nurse who was accompanied by an engineer to install the sensors. The research nurse visited the participants’ homes once a week during the first month and once every month from the second to third month after the sensor installation to conduct in-home interventions and resolve problems using the smart-care system.

### Ethical consideration

The study was conducted following approval by the human subject research section of the hospital ethics committee (Chang Gung Medical Foundation, Institutional Review Board; approval number: No. 201702016B0; No. 201701649B0). Data were collected after the participants provided signed informed consent.

### Measures

#### Qualitative data

Face-to-face semi-structured interviews were conducted by a registered nurse researcher, with a master’s degree in qualitative studies and trained in conducting qualitative interviews. Each interview lasted 30 to 60 min, using an interview guide; interviews were tape recorded with each participant’s permission, and transcribed verbatim for analysis. Sample questions included: “Can you describe a typical day with your spouse/parent/in-law [the care recipient] and what type of care is provided by you? What have your experiences been using the smart-care system? In what ways has the smart-care system been helpful or not helpful in your caregiving [for you or the care recipient]? What has been difficult for you or the care recipient about using the smart-care system.”

#### Quantitative data for family caregivers of persons with dementia

Quantitative data was only collected for caregivers of persons with dementia because, unlike persons recovering from hip-fracture surgery, the trajectory of dementia does not include recovery. Therefore, the quantitative assessments for caregivers of persons with dementia is not likely to be influenced by the care recipient’s recovery.

Preparedness, which is defined as how ready caregivers believe they are for the tasks and stresses of caregiving [4], was measured by the Preparedness Scale of the Family Caregiver Inventory [4, 28, 29]. The 8-item, self-report Preparedness Scale asks caregivers to rate how well prepared they think they are three domains of caregiving: providing emotional support, arranging services needed, and dealing with the stress of caregiving. A final question asks the caregiver to give an overall rating of how well prepared he or she is to care for the care recipient. The total scale score is the sum of the means across all items divided by the number of items [4]. Scores range from 0 to 4; higher scores indicate a greater level of preparedness.

Caregivers’ depressive symptoms were assessed using the 20-item, self-report Chinese-version of the Centre for Epidemiologic Studies Depression (CES-D) Scale [11]. Each scale item on the CES-D is a symptom, which are rated by frequency experienced over the previous 7 days from 0 (less than 1 day), 1 (1–2 days), 2 (3–4 days), to 3 (5–7 days). The summed scores range from 0 to 60, with higher scores indicating more depressive symptoms.

#### Quantitative data for care recipients

We used the 10-item Chinese Barthel Index (CBI) [15] to measure care recipients’ activities of daily living (ADLs), including eating, transferring, grooming, toileting, bathing, walking, climbing stairs, dressing, as well as bowel and bladder control. Cognitive function was assessed with the 11-item Mini-Mental State Examination (MMSE)-Taiwanese version, which assesses an individual’s orientation, memory, common sense, ability to use language, ability to construct thoughts, as well as content of thought, form, and process [32]. Total MMSE scores range from 0 to 30, with higher scores indicating better cognitive function.

### Data collection

Data were collected from family caregivers and care recipients in the participants’ home settings by registered nurses with bachelor’s and master’s degree training in research. Qualitative face-to-face interviews with family caregivers and quantitative data for caregivers of persons with dementia were collected at baseline, and at 1- and 3-months duration of the smart clothes assisted nursing care program. Quantitative data for care recipients was collected at baseline.

### Data analysis

#### Qualitative data

Interviews were transcribed by the nurse researcher who had conducted the interviews. Transcriptions were analyzed by three coders according to Miles and Huberman’s guidelines for three types of codes: descriptive, interpretative, and patterns [19]. Descriptive codes imply little interpretation to reduce the data to key words and phrases. Interpretive codes involving the researcher’s interpretation were based on the research purpose and derived from family caregivers’ perspectives. Descriptive and interpretive codes are first-cycle codes that are used to initially summarize segments of data. Pattern codes, which are more inferential and explanatory, capture patterns appearing in the data to develop themes or categories. Pattern codes, as second-cycle codes, were the outcomes of grouping summaries into a smaller number of sub-themes and themes. In other words, the first-cycle codes were sorted into sub-themes according to their similarities and differences, and further grouped into meaningful themes in the second-cycle coding [22]. These sub-themes and themes were then used to form the coding list, which was revised several times as the coding process progressed and reviewed by the research team.

YJH and SYZ performed the initial coding and YILS re-checked and discussed the coding with them. YJH and SYZ completed courses in qualitative research and training in analyzing qualitative data as part of a master’s program in geriatric nursing. YILS has years of experience as a researcher and instructor in qualitative methods with multiple publications in qualitative studies. The conflicts between coders were resolved through peer discussion with the research team and with YILS. The coding of the data was performed in three stages: the initial coding was performed separately by YJH and SYZ; then the two coders compared their initial findings, which were modified as necessary. The coders accompanied the interviewer to the homes of five family caregivers for consultations, which allowed the coders to become more familiar with the phenomena and to allow family care givers to confirm results of the coded interview data. In the third stage, coded data were checked by YILS. If there were any disagreements at this third stage, codes were discussed with other members of the research team until consensus was reached.

#### Quantitative data

Due to the small sample size (*n* = 7), the non-parametric Friedman test of differences was used to compare repeated measures with post-hoc analysis to determine if there were significant changes in scores for preparedness and depressive symptoms of family caregivers of persons with dementia. Data were analyzed using SPSS 22.0 for Windows.

### Validity, reliability, and rigor

Reliability of the scales for the quantitative findings have been demonstrated in previous studies. The psychometric properties of the Preparedness scale [28, 29] and the Chinese-version CES-D have been validated for Taiwanese family caregivers [18].

Trustworthiness of the qualitative data was established according to the guidelines described by Lincoln and Guba [17]. The researchers’ prior engagement with persons living with dementia and recovering from hip-fracture surgery, and member checking via consultations with five participants to verify the results of the coded data enhanced credibility of the data. An audit trail using peer review of data and reflexive journals from three experts in qualitative studies and dementia care, and peer debriefing with the research group provided confirmability of the data. The rich descriptions from the transcribed interview data were enhanced with field notes, which recorded non-verbal responses of the participants, ensured transferability of the findings to similar family caregivers.

## Results

### Participant characteristics

#### Family caregivers

The 13 family caregivers were comprised of three spouses, eight sons, one daughter, and one daughter-in-law. The mean age of the seven family caregivers of persons with dementia was 64.2 years (SD = 15.7), four were male, five were married, and all were employed full-time. The mean length caregiving time for persons with dementia was 4.4 years (SD = 3.4) with a mean of 7.2 caregiving hours per day (SD = 8.2). All but one caregiver lived with the care recipient full-time. These family caregivers utilized a few community resources such as occupational therapy, family support groups, or dementia activity centers (mean = 0.7, SD = 1.1). The mean age of the six family caregivers of persons recovering from hip-fracture surgery was 66 years (SD = 18.4); five were married and employed full-time; all had a university or college education and lived with the care recipients. The mean length of caregiving was 10.6 years (SD = 8.1), with a mean of 13.5 caregiving hours per day (SD = 11.7). None made use of community support services. Details of the characteristics of the family caregivers are shown in Table 1.

**Table 1:** Demographic and caregiving characteristics of family caregivers (*N* = 13)

Characteristic	Care recipient diagnosis			
	Dementia (*n* = 7)		Hip fracture (*n* = 6)	
Demographics				
Age, years (Mean, SD)	64.2	15.7	66	18.4
Gender (n, %)				
Male	4	57.1	1	16.7
Female	3	42.9	5	83.3
Marital status (n, %)				
Single	1	14.3	1	16.7
Married	5	71.4	5	83.3
Widowed	1	14.3	0	0.0
Education (n, %)				
Elementary school	1	14.3	0	0.0
College/University	1	14.3	6	100
Graduate school	5	71.4	0	0.0
Employed full-time (n, %)				
Yes	7	100	5	83.3
No	0	0.0	1	16.7
Relationship with care recipient (n, %)				
Spouse	2	28.4	2	33.4
Son	3	43.0	4	66.6
Daughter	1	14.3	0	0.0
Daughter-in-law	1	14.3	0	0.0
Caregiving				
Length of caregiving, years (Mean, SD)	4.4	3.4	10.6	8.1
Daily caregiving hours (Mean, SD)	7.2	8.2	13.5	11.7
Number of community services (Mean, SD)	0.7	1.1	0	0.0
Residing full-time with care recipient				
Yes	6	85.7	6	100
No	1	14.3	0	0.0

*Note:* SD = standard deviation

#### Family care recipients

Demographic and clinical characteristics of the care recipients are shown in Table 2. The mean age the seven care recipients with dementia was 85.5 years (SD = 7.5), all were female, three were married and four were widowed; five had an elementary school education. Five persons with dementia had been diagnosed with degenerative Alzheimer’s disease, one with vascular dementia, and one with mixed type. The mean score on the MMSE was 16.29 (SD = 9.4), and ADLs were 75 (SD = 32.0). The mean age of the six care recipients recovering from hip-fracture surgery was 86.5 years (SD = 3.9) years, five were female, and four were widowed. Four had a college/university education. The mean score on the MMSE was 16.3 (SD = 7.6), which did not differ from care recipients with dementia, and the mean score for ADLs was 56.6 (SD = 20.8).

**Table 2:** Demographic and clinical characteristics of care recipients (N = 13)

Characteristic	Care recipient diagnosis			
	Dementia (*n* = 7)		Hip fracture (*n* = 6)	
Demographics				
Age, years (Mean, SD)	85.5	7.5	86.5	3.9
Gender (n, %)				
Male	0	0.0	1	16.7
Female	7	100	5	83.3
Marital status (n, %)				
Single	0	0.0	1	16.7
Married	3	42.8	1	16.7
Widowed	4	57.2	4	66.6
Education (n, %)				
Elementary school	5	71.4	1	16.7
College/University	1	14.3	4	66.6
Graduate school	1	14.3	1	16.7
Clinical characteristics				
ADLs (Mean, SD)	75	32	56.6	20.8
MMSE score (Mean, SD)	16.29	9.4	16.3	7.6
Type of dementia (n, %)				
Degenerative (Alzheimer’s disease)	5	71.4	–	–
Vascular	1	14.3	–	–
Other (mixed)	1	14.3	–	–

*Note*: *SD* standard deviation, *ADLs* activities of daily living based on the Chinese Barthel’s Index score, *MMSE* Mini-Mental State Examination

### Qualitative findings

Interview data indicated the overall experience of participating in the smart-care home healthcare program was that it offered many benefits to both family caregivers that would not typically be available. These benefits were coded into six categories: 1) *monitoring and keeping track; 2) informing the healthcare provider; 3) balancing exercise and safety; 4) motivating exercise; 5) balance work and care; and 6) providing guidance for caregiving.*

#### Monitoring and keeping track

The smart clothing benefited a family caregiver of a person with dementia by providing constant information about the care recipient: *“The smart clothing helps by keeping a record of his [care recipient’s] walking and sleeping.”* (T002-6 M-0042) Another caregiver of a person with dementia said, *“This system provides us information on her [care recipient’s] daily activities and alerts us if anything goes wrong.”*(T005-1 M0061) Another family caregiver of a person recovering from hip-fracture surgery said, “*I think the most helpful thing is that the nurse can remotely monitor her [care recipient’s] condition, so I know how many times she gets up at night, and whether she’s exercising. I think this is very helpful.”* (S007-1 M − 020)

#### Informing the healthcare provider

Caregivers of persons recovering from hip-fracture surgery described the smart-care program as being better at notifying the care recipient’s doctor or nurse about the patient’s recovery at home. One family caregiver said, *“Sometimes doctors don’t really know what is going on at home. I think this system is good because it records her [care recipient’s] condition. It [the sensor feedback] can help doctors understand her situation.”* (S005-1 M-0196) Similarly, another caregiver said, *“This [system] lets the nurse know about her [care recipient’s] condition exactly.”* (S003-1 M-0117)

#### Balancing exercise and safety

Caregivers of persons with dementia commented on feeling less concern about the care recipient’s safety at home. One of these family caregivers said, *“This [program] helps to keep her [care recipient] safe when she exercises. Older people need some reassurance, you know. This helps her feel safe because she knows someone is watching over her.” (T008-3 M-0141)* Similarly, another caregiver said, *“While she walks around, this [system] can alert us if she falls.”* (T005-3 M-0059)

#### Motivating exercise

Caregivers of persons with dementia and persons recovering from hip-fracture surgery reported that the program motivated the care recipient to exercise. One family caregiver of a person with dementia said, “*I told her [care recipient] that because you wear this [smart clothing], the nurse will know whether you’re exercising as they told you to, so she’s more willing to do it.*” (T006-1 M-098) Another caregiver of a person recovering from hip-fracture surgery said, *“It’s [smart clothing] a reminder. She doesn’t like to exercise. Since she began wearing this [smart clothing], she exercises more because other people know whether she exercises or not.”* (S008-1 M-044).

#### Balance work and care

Family caregivers of persons with dementia shared it was easier to be at work knowing that the smart-care system was monitoring the care recipient’s activities, which allowed more of a work-life balance regarding caregiving responsibilities. One of these family caregivers said, *“We have to work; we can’t be there during the day to observe her [care recipient’s] behavior patterns. This [system] provides really useful information for us.”* (T008-3 M-0133) Another caregiver of said, *“I think you guys are great. I feel more relaxed [at work]. I know that if I’m not home and she [care recipient] has an emergency, you would call me. She knows that if something happens, she can push that button.”* (T006-3 M-0122)

#### Providing guidance for caregiving

Family caregivers of persons with dementia and persons recovering from hip-fracture surgery reported receiving information regarding the care recipient’s condition from the SCA home-nursing care program, guided them in adjusting their approach to caregiving. One family caregiver of a person with dementia said, *“The smart clothing records his [care recipient’s] activities. After we found that he actually gets up quite often at night to move around and go to the toilet, my sister bought a potty chair for him to prevent him from falling.”* (T002-6 M-0044) Another caregiver of a person recovering from hip-fracture surgery said, *“Because she [care recipient] was chanting, the system recorded her not moving for 2 hours. I told her not to chant so long at once, or else her gait might get unstable after sitting down too long. I told her to chant for an hour and take a break; if she wants to do more, she can do it later.”* (S003-1 M-0298)

### Quantitative results

Preparedness and depressive symptom scale scores for family caregivers of persons with dementia are shown in Table 3. Mean scores for preparedness at baseline, 1 month and 3 months following the SCA home-nursing care intervention were 2.9 ± 0.93, 2.9 ± 0.86, and 3.0 ± 0.68, respectively; scores at 1- and 3-month follow-up did not differ significantly from baseline. These results suggest these caregivers believed they were “quite prepared” for family caregiving when the study began. For depressive symptoms, the mean score at baseline on the CES-D at baseline was 16.4 (SD = 12.2), indicating mild depressive symptoms. CES-D scores were lower at 1- and 3-months following implementation of the SCA home-nursing care program to 11 (SD = 8.3) and 12.1 (SD = 11.6), respectively. Post-hoc tests indicated significant differences in CES-D scores between baseline and 1-month as well as between baseline and 3-months (*p* < .05), but not between 1- month and 3-months.

**Table 3:** Scores for caregiving preparedness and depressive symptoms of family caregivers for persons with dementia (*N* = 7) at baseline, and 1- and 3- months duration of the SCA home-nursing care program

Scale	Baseline	Duration of SCA program		
		1-month	3-months	
Preparedness	Mean ± SD	Mean ± SD	Mean ± SD	p
	2.9 ± 0.93	2.9 ± 0.86	3.0 ± 0.68	.465
Depressive symptoms	16.4 ± 12.2	11.0 ± 8.3	12 ± 11.6	0.16

*Note*: *SD* standard deviation

### Integration of qualitative and quantitative results

There was a consistency in how the SCA program benefited family caregivers when qualitative and quantitative results were integrated. Quantitative data demonstrate depressive symptoms were lower at 1 month and 3 months compared with baseline measures (pre-SCA) for family caregivers. Benefits demonstrated by the qualitative data varied with family caregivers’ type of care recipient, although there were also commonalities. Caregivers of persons with dementia benefited from sensor monitoring, balancing exercise and safety, and the ability to balance work and caregiving. Caregivers of persons recovering from hip-fracture surgery felt the care recipient’s recovery progress was more accurately transmitted to doctors and nurses. Both groups of family caregivers believed the care recipient was more motivated to exercise. Finally, the greatest benefit reported by both types of caregivers was the inclusion of the homecare nurses responsible for overseeing the program, which allowed for more timely interventions as well as guidance for caregiving. Thus, the benefits suggest the SCA program was helpful for facilitating caregiving.

## Discussion

This mixed-methods study is the first to explore whether a smart clothes-assisted homecare nursing program was beneficial for family caregivers caring for persons with dementia or recovering from hip-fracture surgery. Our findings showed the SCA home-nursing care program allowed homecare nurse researchers to provide timely interventions and guidance to improve caregiving for family caregivers, which provided indirect benefits to care recipients. We also found the SCA home-nursing care program decreased depressive symptoms for family caregivers of persons with dementia, although it did not enhance caregiving preparedness. The lack of impact on preparedness might be because these caregivers already had a lengthy experience as caregivers for persons with dementia, which left little room for improvement. Our study also found the SCA home-nursing care program helped family caregivers balance work outside the home with caregiving and more actively helped family caregivers provide good quality family care by guiding caregiving activities and motivating care recipients to exercise.

Our overall findings are supported by a review on smart-home technologies that monitor older care recipients’ behaviors and activities and use this information to inform caregivers and health care professionals about risk situations so they can take preventive actions [3]. Two major goals of smart technologies that assist family caregivers are helping monitor the care recipient’s condition and providing better connections between caregivers and health care professionals [1]. Our results are consistent with a report that telehealth combined with discharge planning decreased family caregiver burden, improved stress mastery, and improved family function [7]. Our finding that older care recipients recovering from hip-fracture surgery were motivated by the SCA home-nursing care program to exercise echoes findings that increased adherence to rehabilitation by care recipients with hip fracture greatly influences recovery outcomes of persons after hip-fracture surgery [14]. Similarly, information and communication technology systems were found to improve quality of life and safety for older care recipients with dementia [23]. The results of this study are also consistent with a report that informal caregivers of community-dwelling older adults preferred technologies that assisted care recipients with not only ADLs, but also increasing their safety, such as detecting falls, and helping locate them when disoriented [30].

Family caregivers of persons with dementia must not only manage the needs of the care recipients but also their own personal needs. These competing needs of the care recipient and caregiver create high levels of stress and increase the risk for depression and other negative health effects [5]. Having more difficulty in reconciling work and caregiving roles, as well as work inflexibility has been shown to be a predictor of greater role strain and more depressive symptoms [31]. Whereas family caregivers who can balance competing needs while caring for a frail older family member at home are more likely to provide better quality care to the care recipients, have positive caregiving outcomes, and better mental health [27–29]. Balancing competing needs has been shown to mediate the association of caregiving demands with caregiver role strain and depressive symptoms for family caregivers of persons with dementia [18]. This SCA home nursing program facilitated balancing competing caregiving and personal needs for family caregivers of persons with dementia and those recovering from hip-fracture surgery.

Family caregivers believed the SCA program also benefited the care recipients because it helped achieve a balance between exercise and safety, which motivated recipients to exercise on a regular basis. These findings echo other studies demonstrating smart-care systems can enhance exercise efficacy and adherence to rehabilitation [2, 21, 24]. The SCA program provided family caregivers with a sense of security that care recipients would be able to safely participate in exercise activities when the caregivers were unable to provide supervision.

### Implications

Technologies to facilitate family caregiving for persons with dementia or recovering from hip-fracture surgery can be developed to enhance caregiving quality and decrease caregiver burden. Incentives and the flexibility to develop innovative services to support family caregiver needs must be incorporated into long-term care policies.

### Limitations

This study had several limitations. The small sample limited our exploration of how participants experienced our SCA home-nursing care intervention. The sample of caregivers was mixed in terms of recipients, which was comprised older persons with dementia and those recovering from with hip-fracture surgery, which might limit the beneficial findings of this SCA home-nursing care program to specific care-recipient populations. In addition, all but one of the care recipients were female, which may limit the generalizability of this study. In 2020, 47.8% of persons age 15 and older in Taiwan had an education attainment of college and above [20]. The education background of the family caregivers in this study is higher than the general population and might influence the benefits/positive responses of this Smart Clothes-Assisted Home Nursing Care program, and thus influenced the external validity of the study. Future studies on this topic are suggested to use larger samples, use a randomized clinical trial, and focus on a specific care-recipient population. Despite these limitations, this study provides preliminary results to support the benefits of a smart technology-assisted home-nursing care program, thus adding to the current limited knowledge base with little empirical evidence of how smart technology assists family caregivers of older care recipients. The results of this study can also serve as a reference on designing and modifying SCA home-nursing care interventions.

## Conclusions

Our results show that a SCA home-nursing care program increased family caregivers’ knowledge of older care recipients’ condition, informed health care providers about the care recipient’s condition, helped the home are nurse provide timely interventions, balanced care recipients’ exercise and safety, motivated older care recipients to exercise, helped family caregivers balance work outside the home and caregiving, and guided caregiving activities. Therefore, our study findings can be used for a reference for future development in smart-home technology.

## Data Availability

The datasets generated and/or analyzed during the current study are not publicly available due to the principal investigator’s decision to make the data publicly available upon completion of the formal study. To access the data please contact the principal investigator Yea-Ing L Shyu.

## Abbreviations

ADLs: activities of daily living
CBI: Chinese Barthel Index
CES-D: Centre for Epidemiologic Studies Depression
IADLs: instrumental activities of daily living
MMSE: Mini-Mental State Examination
SCA: smart clothes-assisted
SD: standard deviation
